# Cavernous Hemangioma of the Chiasm and Left Optic Nerve

**DOI:** 10.7759/cureus.8068

**Published:** 2020-05-12

**Authors:** Oleksandr Voznyak, Andrii Lytvynenko, Oleg Maydannyk, Olga Kalenska, Nazarii Hryniv

**Affiliations:** 1 Centre of Neurosurgery, Clinical Hospital "Feofaniya", Kyiv, UKR; 2 Centre of Neurosurgery, Clinical Hospital “Feofaniya”, Kyiv, UKR; 3 Department of Pathology, Clinical Hospital “Feofaniya”, Kyiv, UKR; 4 Department of Neurosurgery, Shupyk National Medical Academy of Postgraduate Education, Kyiv, UKR

**Keywords:** cavernous angioma, optic nerve, optic chiasm, surgical resection, case report

## Abstract

Cavernous malformations (CMs) of the optic nerves, chiasm, and optic tract are very rare. This report describes a 26-year-old man who presented with recurring headaches, loss of vision in his left eye, and elevated blood pressure. After being diagnosed with glioma of the chiasm, he was referred to our department. Magnetic resonance imaging revealed signs of a mass lesion of the left chiasmal area, a finding confirmed after transcranial biopsy. In February 2015, he underwent gross total resection of the cavernous angioma of the chiasm and the left optic nerve. Three months later, the patient's vision returned to normal.

The absence of a typical clinical picture and the lack of radiological visualization can hinder pathologic diagnosis. Total microsurgical resection is the optimal treatment strategy for patients with CMs of the chiasm and optic nerve because it usually results in improved vision and long-term benefits. The results in this patient demonstrate the importance of rapid diagnosis and gross total surgical resection of CMs of the chiasm and left optic nerve.

## Introduction

Cavernous malformations (CMs) are the second most common type of vascular formations and account for up to 10% to 15% of all intracranial vascular malformations [1]. CMs of the optic nerves (ONs), chiasm, and optic tract are very rare, occurring in fewer than 1% of patients with brain CM [2]. A "venous angioma" of the optic chiasm, later called a cavernous hemangioma of the ON, was successfully resected in 1978. Through 2005, only 28 case reports or short series (two to four patients) involving 42 patients had been published. An analysis in 2010 described 65 patients with CM of the hypothalamus and optical pathways, with an update in 2014 including 70 patients [3,4]. The latter remains the largest review to date of patients with CM of the ONs and chiasm. 

Three patterns of clinical onset have been described: acute (chiasmal apoplexy), subacute, and progressing. Acute CM of the ONs and chiasm is characterized by sudden blurred vision, headache, nausea, and retroorbital pain [5]. The other two patterns include the gradual loss of vision, similar to that observed in patients with suprasellar tumors and ON gliomas. Surgical strategies used for CM of visual pathways include gross total resection, subtotal resection, and resection following by optic canal unroofing biopsy with decompressive evacuation of the hemorrhage, pure biopsy, and biopsy with radiation [6]. Surgical resection is the preferred treatment for recovery of ON function and elimination of future risks of hemorrhage [7]. Clinically and radiologically, ON cavernomas may mimic other focal lesions of the ONs, including inflammations, gliomas, and demyelination. Thus, despite their rarity, optic CM should be considered in the differential diagnosis of patients with these pathologies, especially ON gliomas [8]. The present study describes a patient with CM who was presented clinically and radiologically as a glioma of the left ON and chiasm.

## Case presentation

A 26-year-old man was admitted for recurrent headaches, loss of vision in his left eye, and elevated blood pressure. His loss of sight had been gradual, manifesting for over six months before hospitalization. He also exhibited episodes of inadequate behavior with some aggression. The patient was conscious at admission, and no other meningeal or focal neurological symptoms were found. The patient consented to publication of his protected health information.

**Figure 1:**
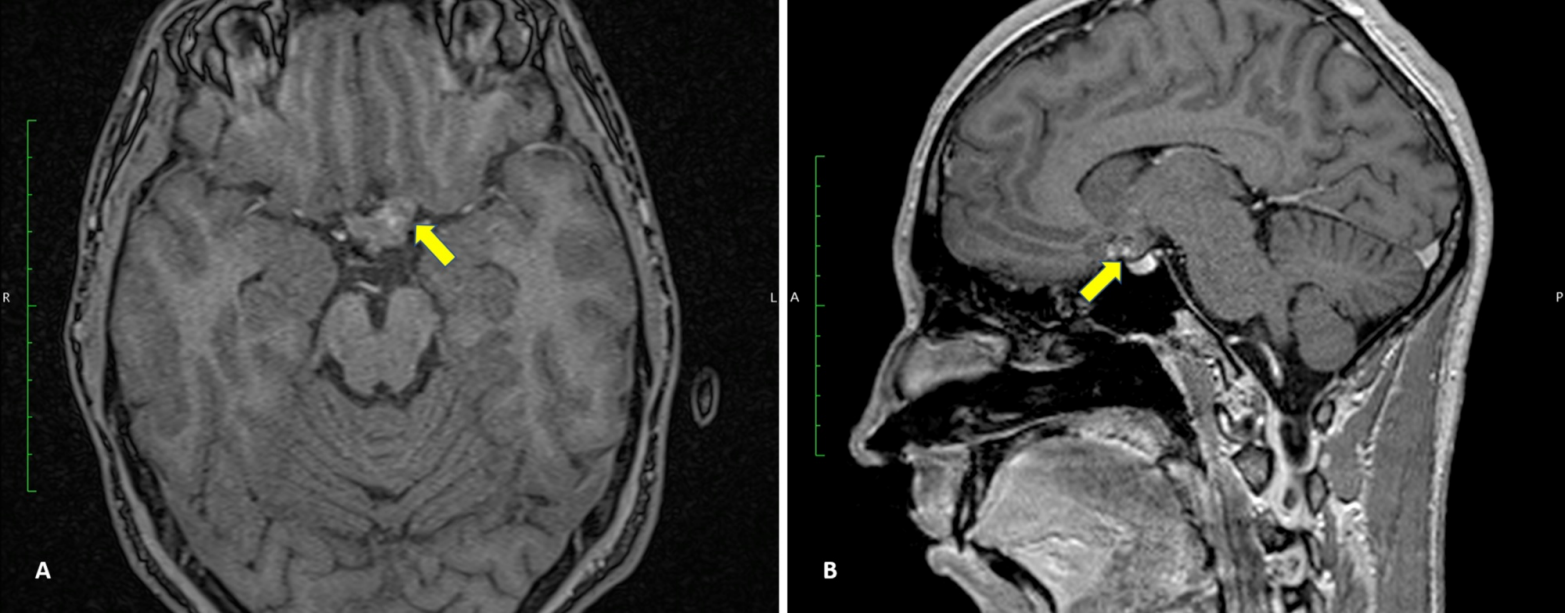
Preoperative (A) axial and (B) sagittal MRI. Hyperintense signal on T1-weighted MRI showing a mass lesion occupying the chiasm and left optic nerve.

His right and left eyes had logarithm of the minimum angle of resolution visual acuities of 1.0 and 0.2, respectively. T1-weighted magnetic resonance imaging (MRI) showed a hyperintense signal mass lesion occupying his chiasm and left ON (Figure 1). These findings led to a diagnosis of high-grade glioma of the chiasm and left ON, or CM.

Surgery was performed with the patient in the supine position, with his head slightly elevated and turned to the right side about 30 degrees. A left frontolateral craniotomy was performed to optimize the approach to the chiasm and left ON. Fenestration of the basal cistern and aspiration of cerebrospinal fluid yielded a relaxed brain with satisfactory gravity retraction. The left optic canal was unroofed, and several blood clots at different stages of resorption were evacuated through the longitudinal dissection of the ON. Minimal coagulation currents were applied to occlude the feeding vessels with their sharp sections. Resection was estimated to be gross total (Video 1).

**Video 1:** Gross total resection of a cavernous hemangioma of the chiasm and left optic nerve.

**Figure 2:**
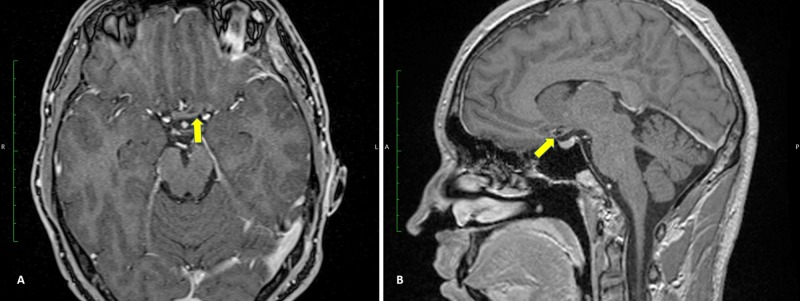
Postoperative (A) axial and (B) sagittal MRI.

There were no surgical complications, and MRI performed six months later showed no signs of CM recurrence (Figure 2). The patient was discharged after seven days, and his vision returned to normal three months later. He is still in touch with us and has reported no signs of visual deterioration or recurrent bleeding.

**Figure 3:**
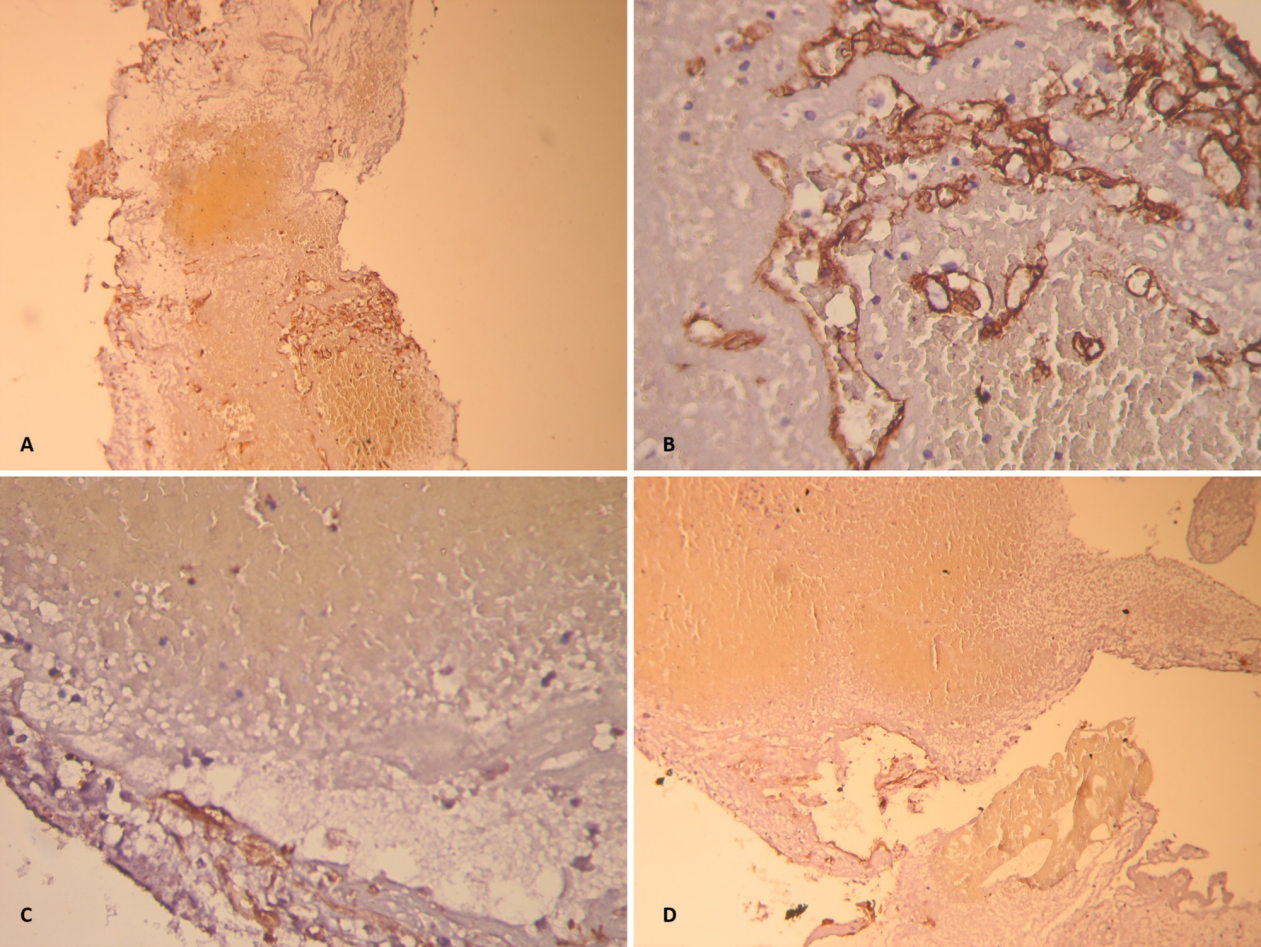
Microscopic structure of the resected cavernous malformation, showing heterochromous thromboses. (A) Positive expression in the endothelium of small thin-walled vessels CD34 (clone QBEnd/10); 20-fold magnification. (B) Positive expression in the endothelium of small thin-walled vessels CD34 (clone QBEnd/10); 40-fold magnification. (C) Actin, smooth muscle Ab-1 (1A4) - positive expression in myoepithelium of enlarged thrombotic vessels. (D) Expanded, thrombotic thin-walled CD34 (clone QBEnd/10) - positive vessel, lined with cohesive endothelium.

The microscopic structure of the resected CM with heterochromous thromboses is shown in Figure 3.

## Discussion

Cavernomas are vascular malformations that are usually benign. They consist of endothelial cell-lined, dilated vessels (caverns) packed together without intervention to the neural parenchyma [9]. Cavernomas, the most common type of angiographically occult malformations, occur in 0.3% to 0.5% of patients and account for 10% to 15% of all vascular malformations [10]. In contrast to arteriovenous malformations, CMs are characterized by low blood flow and cannot be visualized by angiography [11]. They can occur throughout the central nervous system, most frequently in supratentorial compartment (70% to 80%), followed by the infratentorial region (15%), spinal cord (5%), and most rarely, cranial nerves [12]. Although optical pathways are the most frequent locations of CMs in cranial nerves, fewer than 100 such patients have been described worldwide [13,14]. 

The most common patient concern is a loss of vision (98%), followed by headache or retroorbital pain (60%). Symptoms are acute in 58% of patients, subacute in 15%, and progressive in 26% [3]. Unlike CM, optic gliomas are often characterized by slow unilateral loss of vision, protrusion of the eyeball, decreased color vision, swelling or atrophy of the optic papilla, or strabismus. Nevertheless, malignant gliomas of the ON can manifest as a sudden loss of vision in 70% to 84% of the adult patients [15].

Neuroimaging has shown that 88% of CMs of the optic tract are characterized by heterogeneous features with mixed-signal intensity. T2-weighted imaging has shown a hypointense peripheral ring (hemosiderin) in 60% of patients [16]. Cavernomas are also characterized by minimal or no gadolinium enhancement. Neoplasms of the optic pathways can present as thickening of the nerves with increases in their diameter [3]. Typical MRI findings in patients with ON glioma include isointense and hyperintense masses on T1-weighted images and hyperintense masses on T2-weighted images with homogeneous contrast enhancement [4]. Although hemorrhage foci on MRI may be indicative of CM, it may be difficult to distinguish CM from ON glioma [3,4].

CM biopsy and radiation resulted in the stabilization of vision in one patient [17]. Although stereotactic radiosurgery may also be effective, no study to date has reported the success of radiosurgery for these lesions [17]. Retrospective reviews have reported higher rates of visual preservation and improvement after complete lesion removal [4]. Microsurgical techniques have been reported to be the only efficient care modality in symptomatic patients [6]. Observation strategy could be considered if there were no signs of tumor's hemorrhage and vision loss.

Gross total resection should be considered the treatment of choice, as any remaining parts of a malformation can progress and re-bleed, placing the patient at risk of disability. Moreover, reoperation may be more difficult and dangerous due to adhesions and the risks of unintentional ON injury [18]. Biopsy of ON lesions is not recommended because of the risks of bleeding and visual deterioration [19]. Patients with mass lesions of the chiasm should be treated surgically because the complete surgical removal of CM leads to patient recovery and prevents re-bleeding.

## Conclusions

CMs of the visual pathways are exceedingly rare. The absence of a typical clinical picture and the lack of radiological visualization can make pathologic diagnosis difficult. The optimal treatment of patients with CM consists of total microsurgical resection because it usually results in visual improvement and good long-term results. Also, resection can provide pathological verification, leading to a more specific therapy.
